# People and sites as community resources for preventing and managing chronic health conditions: A conceptual analysis

**DOI:** 10.1371/journal.pgph.0003415

**Published:** 2024-07-26

**Authors:** Prachee Agrawal, Seye Abimbola

**Affiliations:** Faculty of Medicine and Health, The University of Sydney, Sydney, New South Wales, Australia; George Washington University Medical Faculty Associates, UNITED STATES OF AMERICA

## Abstract

The prevention and management of chronic non-communicable diseases (NCDs) require community-based efforts–especially as their burden grows, and recognition of the need for engaging diverse stakeholders in care grows. The aim of this conceptual analysis was to categorise existing community resources (people and sites) used to support such efforts, the mechanisms by which they work, and the conditions that constrain their effectiveness. We developed an initial framework for categorising community resources. We then used a sample of the literature– 24 studies from 12 countries–to refine and enrich the framework. We identified seven categories of sites (’where’: House, Meeting place, Community health centre, Primary health care centre, Mobile clinic, Pharmacy, and Hospital) and ten categories of people (‘who’: Community Health Worker, Nurse/Midwife, Health educator, Physician, Primary Health Care Worker, Peer Group, Navigator, Pharmacist, Cultural Advisor, Family caregiver). We identified eight mechanisms through which those resources improve NCD prevention and management: Context specific engagement, Personalised and integrated care, Community health worker led knowledge dissemination, Social support through family and/or friends, Extending the reach of the health system, Social support through peer groups, Task shifting, and Training of health workforce. We identified two broad categories of constraints on these mechanisms: (i) health system barriers such as inadequate workforce, training, coordination and engagement; and (ii) socio-economic, political, and cultural barriers to care. The conceptual categories (of people and sites as resources, the mechanisms through which they work and the contextual constraints on their effectiveness) identified in this analysis may be useful in further analysing current approaches in NCD efforts using community resources, in informing the development of community-based efforts, and in exploring the commonalities and transferable insights between different locations or settings around the world and between different efforts to prevent and manage NCDs within communities.

## Introduction

Leveraging the relationship between health systems and the communities they serve, can be key to advancing global heath equity. Physical spaces within physical communities such as the house, meeting places, primary healthcare centres or mobile clinics serve as vibrant, collective and community oriented settings for health-related human-to-human interaction and knowledge-sharing, even in the most remote and distant communities around the world. Exploring, studying and documenting the impact of community approaches to manage chronic conditions is as crucial as focussing on advancing ground-breaking technologies to prevent and manage these conditions. The word ‘community’ was derived from the Latin word ‘communitas’ which means ‘the same’. Underpinned by similar experiences and shared knowledge, social support and human connection, community approaches in care can fill a crucial gap in improving the quality of life of people with lifelong disease, by creating a feeling of belonging, being accepted, cared for and supported by people and the health system.

The growing burden of chronic diseases is largely a result of the exposure of populations to health damaging circumstances (physical, psychosocial, socioeconomic, political and cultural), including industrialisation and manipulation of global food markets that impact dietary choices [1, 2]. These exposures play out within communities, influencing individuals’ choices that impact nutrition, health and the consequences of ill-health. As a result, prevention and management of chronic disease require interventions that operate through the community. Chronic diseases are often lifelong. Having a physical community to share experiences and resources of support with, can improve health outcomes [3–5]. Identifying, classifying and understanding how various health resources operate in their context and settings, can help us better leverage the benefits of community based interventions.

The four main types of NCDs are cardiovascular disease (CVD), diabetes, cancers and respiratory diseases [6]. While some of the risk factors for NCDs are non-modifiable e.g., genetics, other risk factors are modifiable e.g., smoking, unhealthy diets, physical inactivity, alcohol use, and largely emerge through the influence of interrelated determinants of health such as the physical, psychosocial, socioeconomic, political and cultural context of individuals and communities [7]. NCDs are a complex set of health problems which require intersectoral, multifaceted, systems approaches to think about the broader perspective [8] and to make sense of the complex interactions between people, health systems, organisations, resources and various other actors influencing NCD prevention and management [9]. Community based participatory interventions are one such systems approach to NCD prevention and management. Every community is a complex system with multiple interacting parts and peculiar contextual factors, requiring engagement from diverse actors within and outside the community to protect, restore and improve health [7–9]. Clarity around types of community resources, interventions, settings, their mechanisms and barriers can inform systems thinking approaches for NCDs.

This analysis focusses on people and sites. Health interactions typically begin in the community, where service delivery is driven by people of the formal and informal health system; and members of the community in specific sites of the community. These sites reflect the context of the community framed by their social, economic, political, environmental, geographical and cultural conditions. The actors in turn function within the social, cultural, economic and political norms framing health in the context of their community. As such, community based NCD prevention and management are driven by people; people who are serving as resources of the formal and informal health system, and people who participate in community health initiatives and in various other community organisations. While community based interventions are increasingly conducted across the world, there is limited documentation of the evidence and experience of how these interventions make use of existing community resources–as people or as sites within the community–to improve health [10].

Given that different community-based NCD interventions in diverse settings use community resources (people and sites), it is important to have a systematic understanding of the community resources and contexts in which these diverse and numerous interventions get implemented. Especially because little is documented about what community resources (people and sites) are best enabled in which physical setting and which type of community intervention could best fit a community within its context and constraints. While the role of community resources in chronic disease prevention and management is studied in interventions across the world, optimising their use requires that insight on different community resources are systematically organised to better interpret and apply different interventions in different contexts, using various community resources.

The aim of this conceptual analysis was to identify and categorise the numerous community resources used in community based interventions/programmes/actions, thereby bringing clarity to the enabling mechanisms of these interventions and the contexts in which they work.

## Methods

This conceptual analysis was conducted in six stages:

### Stage 1: Developing an initial framework

**Fig 1:**
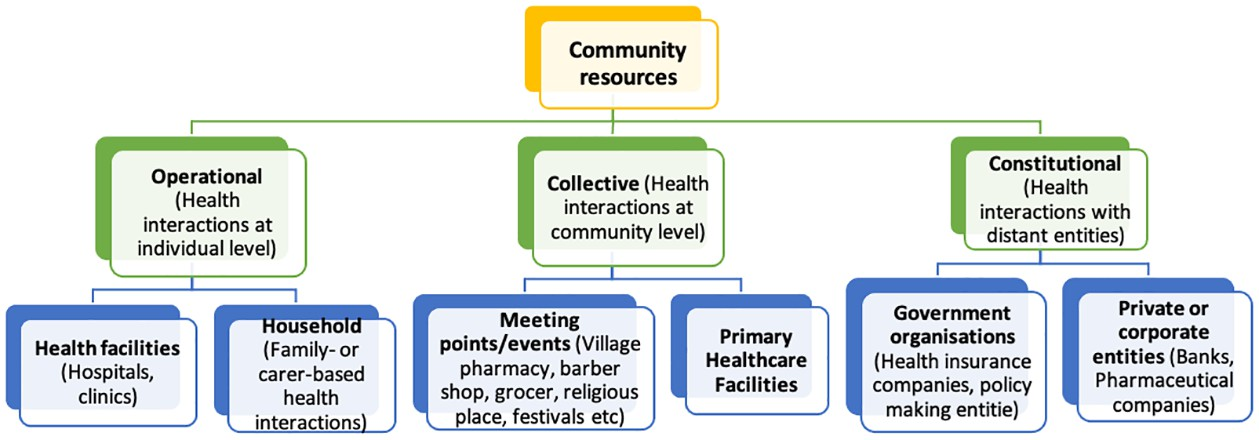
Developing the initial framework.

Development of an initial framework to categorise sites within a community that may constitute resources for chronic disease prevention and management. The initial framework was developed based on discussions between the authors; and grounded in their lived and professional experience of chronic disease prevention and management (in India and in Nigeria), and on a scan of the literature (Fig 1). The sites were initially categorised as operational, collective and constitutional based on the type of interaction for health service delivery in a community and whose remit it is to govern or convene those interactions [11]. Operational sites included households, hospitals and clinics where health interactions occur between the individual and health system. Collective sites were community meeting points such as barber shops, grocery stores, local pharmacy, primary healthcare centres etc where interactions take place on a community level involving individuals and health system representatives. Collective sites also included temporary sites of meeting during festivals and events which serve to facilitate health related information/promotion among people on a large scale. Constitutional sites included government regulated organisations which may be public or privately owned but which are not primarily for the purposes of health care delivery or community convening. These sites include policy departments, banks, pharmaceutical companies, government offices etc where decision making impacts healthcare. The figure below summarises the initial framework.

### Stage 2: Identifying a sample of the literature

Formal search of the literature to identify a sample of the literature describing community based interventions/programmes/actions for the prevention and management of chronic disease. The aim was to test and refine the initial framework in stage 1. The search included identifying qualitative and quantitative studies on community based interventions. Our aim was not to be exhaustive, but instead to identify a sample of the literature. Hence, we limited the search to two of the common forms of NCDs (type 2 diabetes and cardiovascular disease). Medline electronic database was used to search for English language studies on prevention, treatment, management and maintenance of diabetes and CVD in the last three years from February 2020 to February 2023, across all countries. The studies specifically were limited to interventions or descriptions or programmes that were based on human to human interactions in a physical space–as such, telehealth and text message based interventions were not included. The combination of keywords used for the search was as follows:

[Community Health Services/ or community-based resources.mp. or Social Support/ OR Community Health Services/ or community based resources.mp. OR Community Participation/ or village community*.mp. OR community link*.mp.OR Community Participation/ or community asset*.mp. OR familial support.mp. OR Rural Health Services/ or community connector*.mp. or Health Services Accessibility/] **AND** [hypertension.mp. or Hypertension/ OR blood pressure.mp. or Blood Pressure/ OR Diabetes Mellitus, Type 2/ or Diabetes.mp. or Diabetes Mellitus/] **AND** [Management of.mp. OR treatment of.mp. OR Prevention.mp. or Primary Prevention/ or Secondary Prevention/]

**Fig 2:**
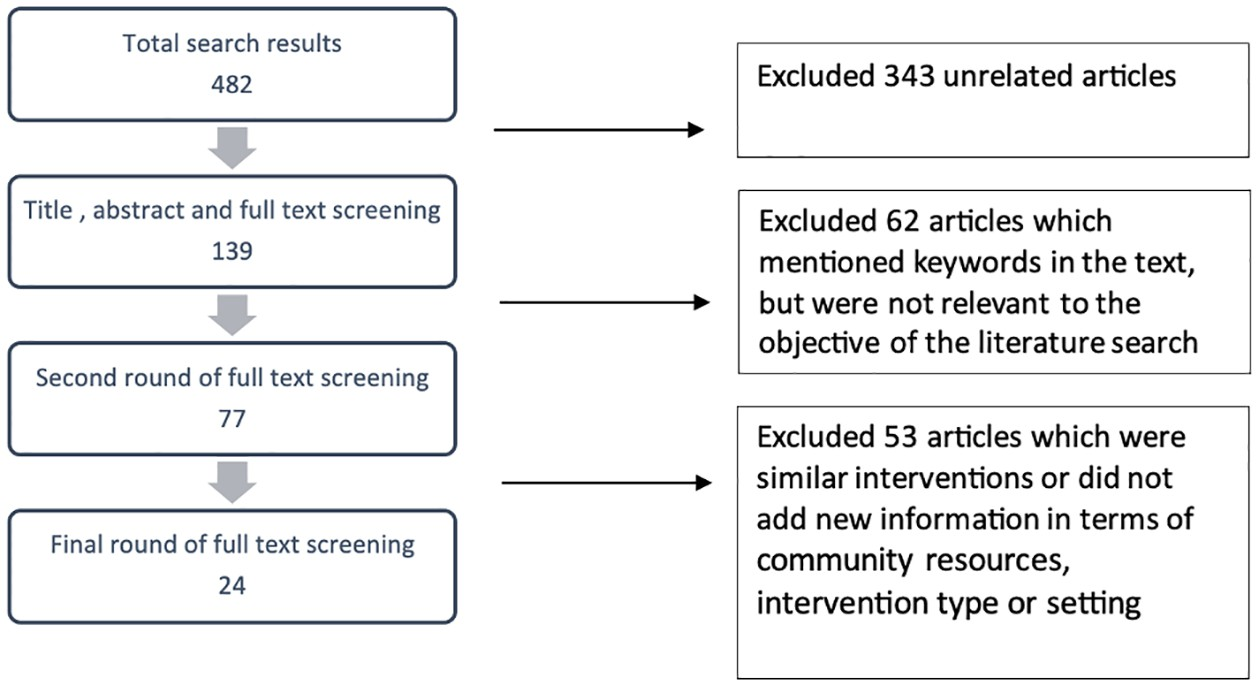
Flowchart of the study selection for literature review.

After multiple rounds of finalising keywords, the final query on Medline yielded 482 studies. We removed 343 studies which were unrelated to our topic. These included topics such as primary healthcare readiness, economic evaluations, tele-education studies, protocols, scoping reviews and systematic reviews. We filtered out another 62 articles after title, abstract and full-text reading. These studies mentioned community resources as per the keyword search but did not provide specific details required based on our objectives. We arrived at 77 studies which were related to the purpose of our literature search and finally selected 24 studies (Fig 2) after removing articles reporting similar community interventions, programmes or descriptions or did not add to any new information in terms of community resources, intervention type or setting. We conducted three rounds of full text readings in the process while recording the filtered articles in an Excel sheet.

### Stage 3: Refining the initial framework

**Fig 3:**
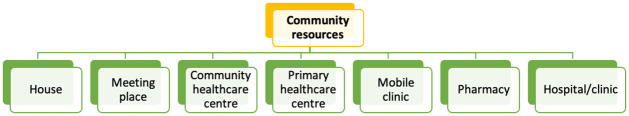
Refining the initial framework.

Refining the initial framework by re-categorising sites, drawing on the examples identified in the literature sampled via the formal literature search. The initial identification of sites included settings where the physical interaction between humans for health service delivery was not applicable and therefore were omitted from the final categorisation. These sites were government departments, banks, private and public companies and other formal regulated organisations. From the formal literature search, three new categories of sites were identified and added to the list–community healthcare centre, mobile clinic and pharmacy. The final list comprised seven categories of sites–house, meeting place, hospital, community healthcare centre, primary healthcare centre, mobile clinic and pharmacy (Fig 3). Except for the house and hospital (operational), all the final sites reflected health delivery through collective interaction in community based settings, therefore the final categorisation was simplified by maintaining only one level of site based categories and eliminating the broad classification of operational, collective and constitutional interactions.

### Stage 4: Further refining and adding to the framework

**Fig 4:**
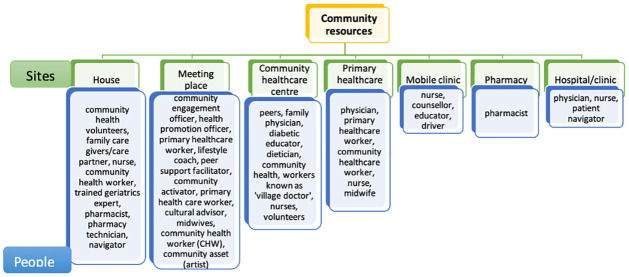
Further refining and adding to the initial framework.

Refining the initial framework (Fig 4) by identifying an additional set of community resources (i.e., people) drawing on the examples identified in the literature sampled via the formal literature search–which were then superimposed on the previous category of community resources (i.e., sites). Sites of healthcare delivery were a key aspect in all articles from the literature search. However, every site had people with distinct roles, contributing to the success or failure of the community based interventions for chronic conditions. We included people as an additional set of community resources to our conceptual analysis for an in-depth perspective on the mechanisms and barriers that impact NCD based community interventions and action.

### Stage 5: Identifying mechanisms and barriers

For each site, we identified contextual conditions that enable or constrain the ability (of sites and people) to effectively deliver or receive community based interventions/programmes/actions for the prevention and management of chronic disease. These contextual conditions were identified by reading and documents the factors identified in each study (that is, as a result of the analysis conducted in the study) or attributed by authors of a study to explain the intervention’s effectiveness or lack thereof (as context or background of the study or in contextualising the results of the study).

### Stage 6: Linking the framework to the ecological model

After creating the framework on community resources (sites and people) and identifying mechanisms and barriers from the sampled literature, we linked them to the ecological model on health promotion [12]. The broad spheres of influence in the model (intrapersonal, interpersonal, institutional, community, policy) when mapped to various community resources, mechanisms and barriers, provides a nuanced perspective on the interplay between elements of the context (community resources and barriers) and elements of the mechanisms (Interventions/descriptions/actions).

## Results

The sample literature consisted of 24 studies from twelve countries–USA (9 studies), Australia (3 studies), China (2 studies), Ghana (2 studies), Canada (1 study), India (1 study), Jordan (1 study), Kenya (1 study), Nepal (1 study), South Africa (1 study), Sri Lanka (1 study), and Sweden(1 study). Almost sixty percent (14/24) of the studies were conducted in high income countries (HICs) with USA reflecting the highest number of studies. Of the articles (10/24) from middle and low income countries (LMICs), China and Ghana displayed more studies compared to others. Majority of the articles were qualitative studies (30%) followed by equal number of mixed method studies, quantitative studies, pre-post analyses and two randomised control trials. The sample included a diverse range of studies on community based interventions in first nations peoples [13, 14] (2 studies), refugees [15] (1 study), older populations [16] (1 study), women [17–19] (3 studies), socially and economically disadvantaged groups (2 studies), rural [20–27] (8 studies) and urban populations [28–34] (7 studies).

Across the 24 studies selected, we identified four sets of themes to characterise the community interventions/programmes/descriptions/actions–‘who’ (people as resources—involved in the interventions), ‘where’ (site as resources of the interventions; or the location where the intervention was implemented), ‘mechanism’ (how the nature/mechanism of the community intervention/action/programme itself acted as an enabler for the prevention and management of NCDs) and ‘barrier’ (the external factors or constraints that hindered communities from accessing healthcare and/or factors impacting the intervention negatively).

Ten categories of community resources (people) were identified. The people who constituted community resources functioned as part of both the formal and informal health and care systems. Except for nine studies [18, 23, 25–28, 30, 31, 34], all others involved more than one category of people as community resources in the interventions and displayed coordinated working between the health system, informal actors in the health system and different community resources.

Various interventions observed across the studies were grouped under eight broad categories based on their nature and the mechanism by which they enable better NCD prevention, treatment and management in communities, thereby constituting the ‘mechanism’ category of our analysis.

**Fig 5:**
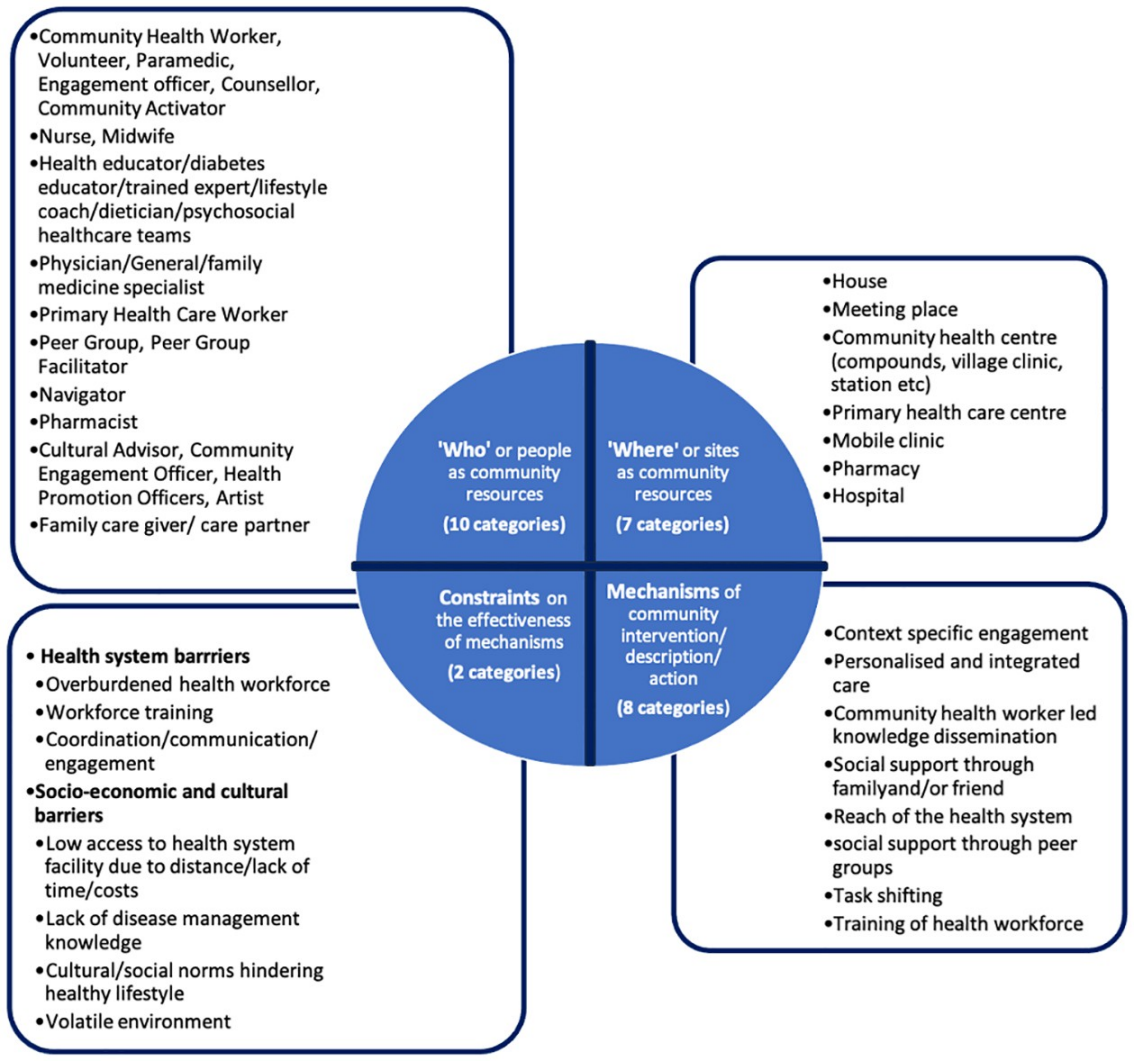
Identified community resources, settings, mechanisms and barriers from the selected studies.

Seven sites of community actions/interventions/programmes were observed ranging from households to mobile clinics and pharmacies. Twenty out of 24 studies (83%) were implemented in households, meeting places, community health centres and primary health care centres. The other important sites identified were mobile clinics, hospital/clinic and pharmacy. Two broad categories of challenges/barriers were identified throughout the review–(i)health system barriers such as lack of adequate healthcare workforce, training of healthcare personnel, coordination and engagement challenges; and (ii)socio-economic determinants, cultural barriers and knowledge gaps in disease management (Fig 5).

This conceptual analysis is organised according to sites as resources, with a focus on how people as resources function in for preventing and managing chronic conditions, the enabling ‘mechanisms’ (community interventions/actions/programmes) of their functions and the contextual factors that constitute barriers to their function as resources for preventing and managing chronic conditions.

**Fig 6:**
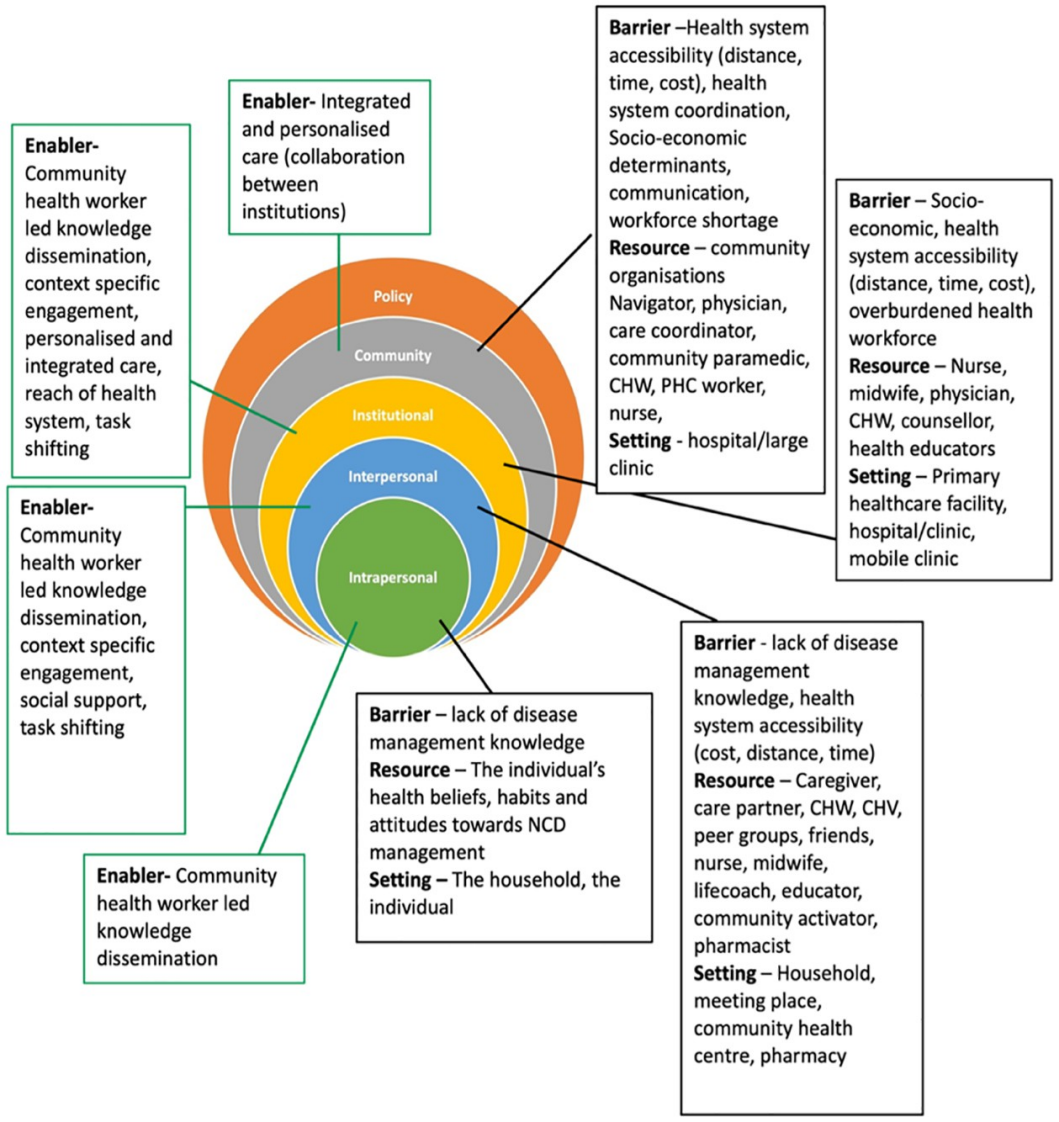
Mapping of community resources, settings, interventions(enabling mechanism) and barriers to the ecological model.

In line with the ecological model, we placed the contextual factors (available community resources and barriers) side by side with identified NCD community interventions and strategies (Fig 6) to focus attention on the nature of community resources and barriers operating under various spheres of influence (intrapersonal, interpersonal, institutional, community, policy) to enable an all-inclusive perspective on community based intervention approaches.

### House

Nine of the 24 studies [16, 19, 21, 24, 27, 31, 32, 34, 35] mentioned the ‘house’ as a setting for community based interventions/action. Of these nine studies, four were conducted to investigate exclusively home based NCD prevention and management, while the rest of the five studies included other settings along with the house e.g., community health centres and meeting places. The house served as a readily accessible setting to implement community interventions through local, well trained community health resources in building better health literacy and NCD management among carers and patients. The community action being facilitated at the household ensured that families caring for people with chronic illness are well trained to manage the disease at home and reduce dependency on secondary healthcare [36].

#### People as resources involved

The main community resources (people) active in NCD prevention and management at the household setting were community health workers/navigators/volunteers, family care givers, geriatrics experts and pharmacists. These community resources supported NCD management at individual household levels to address socio-economic and knowledge based challenges. However, they themselves were limited by the health system mainly through inadequate training, being overworked and low quality supervision.

#### Enabling mechanism

The key mechanisms (interventions/programmes/descriptions) that aided better NCD management in this setting were context specific engagement and community health worker led dissemination of knowledge. Personalised care, integrated care and social support through family and friends also acted as enabling mechanisms in disease management in this group. Coordination among different stakeholders such as CHWs, community health paramedics (CHP), navigators, clinical staff, community organisations and care givers were crucial aspects of successful integrated care delivery. Enhanced training of health workforce and culturally secure dissemination of knowledge helped build a strong partnership between the health system, participants and community based organizations.

#### Constraining barriers

**Fig 7:**
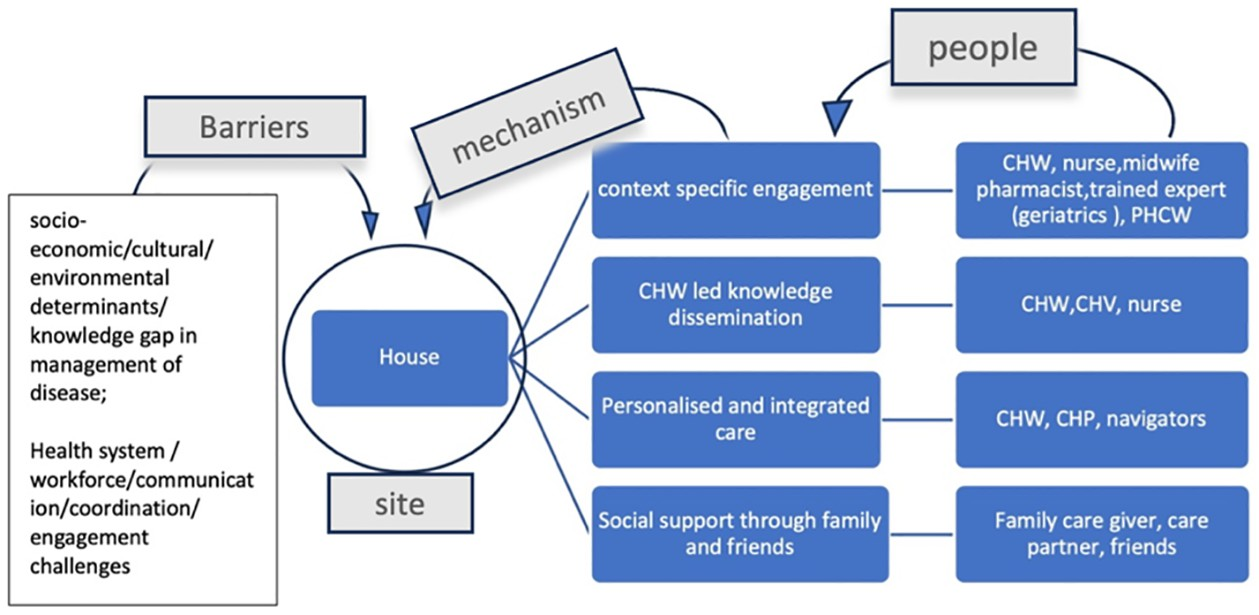
Community resources, enabling mechanisms and barriers associated with the ‘house’.

The barriers associated with NCD prevention and management in house based settings were of two types. The first being barriers related to socio-economic conditions and lack of knowledge on NCD management. The second being health system barriers related to untrained and/or overburdened health workforce, low quality of supervision of health workers visiting homes, community health workers not well integrated in the health system, inadequate financial support of health workers and insufficient use of health resources committed to health prevention and management programmes. These barriers either hampered the implementation of effective NCD prevention and management in the household or restricted access to NCD prevention and management facilities for socio-economic reasons. The main challenges faced here were related to clarifying misconceptions and challenges that primary care givers faced in managing the complexity of chronic illness as well as socio-economic factors such as communication barriers, limited affordability of nutritious food such as fruits and vegetables, minimal time to pursue active lifestyle amidst family demands, transportation and housing, health literacy and racial/ethnic disparities in the management of NCDs (Fig 7).

### Meeting place

Nine of the 24 studies [14, 17–20, 27, 29, 35, 37] mentioned ‘meeting place’ as a setting for community based action on NCD prevention and management. Meeting places may be permanent or temporary venues for community interventions/actions. Permanent venues identified include community clinics, primary healthcare centres, town camps, YMCA centres, churches, pharmacies, public libraries, recreation centres and local care centres [14, 17, 18, 37]. Temporary meeting places included funfairs, carnivals, travelling dramas and local venues where participants and CRs decided to meet in their vicinity [19, 29]. The local settings used as meeting places supported NCD prevention and management interventions through their better accessibility, social engagement and culturally secure knowledge dissemination.

#### People as resources involved

The community resources (people) active in NCD prevention and management in meeting places were CHWs, midwives, lifestyle coaches, navigators, cultural advisors(provide cultural insights in interventions for indigenous populations), community engagement officers (facilitate involvement and participation of the community by raising awareness about the intervention), health promotion officers, peer support facilitators, community activators (trainers of peer support facilitators), local artists and PHCWs. Meeting places had the most diverse settings and categories of people as community resources. These settings were common in interventions targeting specific populations for NCD prevention and management such as Aboriginal community adults, Hispanic communities, pregnant women, Black American women with diabetes and Aboriginal children etc.

#### Enabling mechanism

The interventions based on context specific engagement, social support through peers, personalised and integrated care and CHW led knowledge dissemination enabled useful NCD management in these settings. Meeting places also enhanced reach of the health system through CRs, enabling more people to participate in NCD health initiatives. Peer support groups at meeting places reduced feelings of isolation through community engagement among people undergoing similar health conditions. Programmes driven and owned by indigenous populations facilitated culturally appropriate interventions for indigenous groups; and engaging household decision makers alongside community stakeholders further helped families understand ways of managing chronic illness at their end. Integrated care approaches involved community-clinical linkages, which are unique partnerships between healthcare system and community based organisations to assist and support people with chronic diseases in various ways such as food delivery, medicine delivery etc.

#### Constraining barriers

**Fig 8:**
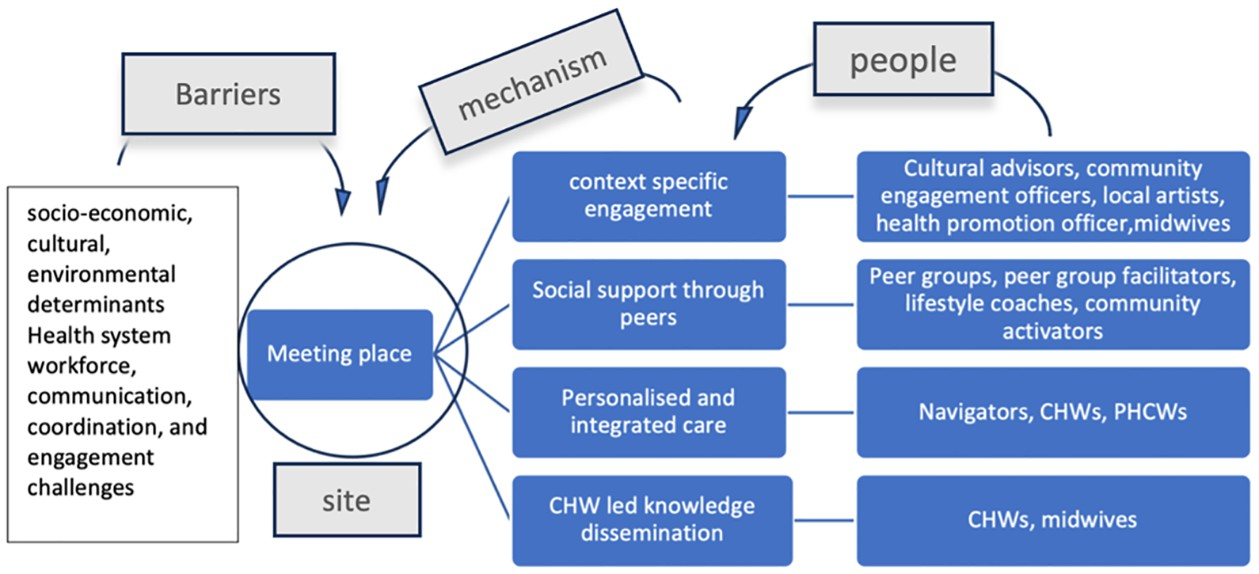
Community resources, enabling mechanisms and barriers associated with the ‘meeting place’.

Six out of the nine studies featuring meeting place as resource (site) had barriers related to socio-economic conditions, cultural/environmental determinants and social norms that reduced access to NCD management and knowledge. For example, in one study American Black women participating in the national diabetes programme stated unavailability of healthy eating options during community functions and gatherings as an environmental/community barrier to continue with healthy diet and maintain individual health preferences [18]. Three out of the nine studies mentioned health system barriers related to coordination, communication and conflicting personalities among different people (as community resource) leading to reduced engagement in NCD management interventions, insufficient use of community resources/funding provided for programmes and not integrating CHWs well within the health system as a valued member of the care delivery team (Fig 8).

### Community health centre

Five out of the 24 studies [13, 21, 25, 30, 32] mentioned community health centre as the setting for implementing community based interventions for NCD prevention and management. Three interventions involved one or more setting (house and PHC) beyond the community centre. Most of the community centres were known by different names in different countries e.g. ‘compounds’ in Ghana, ‘stations’ in Urban China, ‘village clinics’ in rural China, and ‘community clinic’ in Canada. The role of community health centres as a setting for NCD prevention and management is crucial as they not only serve the purpose of a meeting place but in many cases, they also act as centres for training of informal health care providers by the primary health care personnel and act ‘close to ground’ by focussing on social determinants of health. From a governance perspective, this setting was unique, as it involved community members, community organisations, formal and informal healthcare workers working together, reflecting a ‘close to ground’ mechanism thereby reducing health inequity by increasing community involvement in health related decision making [38].

#### People as resources involved

Community health workers/paramedics/volunteers, diabetes educators, peer educators, nurses, village doctors (informal providers), dieticians and family physicians were active in this setting. In some studies, national health programmes driven by primary healthcare resources operated out of community health centres for better reach of screening services and NCD awareness activities e.g. the community health and planning services (CHPS) programme in Ghana and the public health service programme (PHSP) in China utilised community centres alongside PHCs for implementing screening and care packages for diabetes and cardiovascular disease.

#### Enabling mechanism

In some studies, insufficient training resulted in irregularities of the functioning of the community health centre workforce (CHPS, Ghana). In other studies, this setting enabled better trained informal health work force due to regular training of village doctors by the primary healthcare personnel (PHSP China). Key mechanisms in community health centres were CHW led knowledge dissemination, integrated care and context specific engagement. An interesting example of peer learning with the involvement of the local clinic is the concept of ‘shared medical appointments’ [13], where a community clinic conducts a peer learning session as well as NCD management education through a common shared medical appointment among members of the same community. Along with peer interaction, there is also the involvement of a health educator thereby allowing for culturally secure and open conversations around health management. This form of peer group engagement is especially helpful in rural communities and Indigenous communities who prefer collective health initiatives [39, 40].

#### Constraining barriers

**Fig 9:**
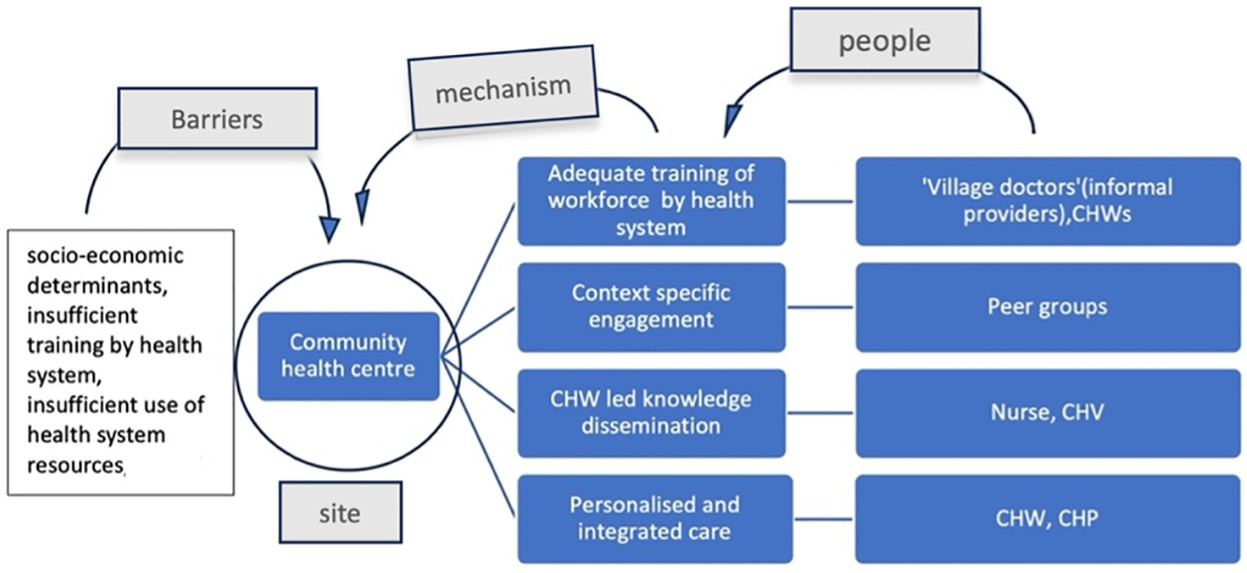
Community resources, enabling mechanisms and barriers associated with the ‘community health centre’.

Key barriers disabling NCD prevention and management action in this setting were lack of trained health workers, overburdened nurses and CHWs, lack of official support for community health workers and inadequate financial incentives, CHWs viewing their primary role in infectious diseases and maternal health rather than NCD management. Among socio-economic barriers, insufficient access to nutritious food and non- availability of transportation were observed. In one study of this setting English language was reported as a barrier to understanding health related information (Fig 9).

### Primary healthcare centre

Primary healthcare centre as a setting for NCD prevention and management was observed in four [15, 17, 26, 30] of the 24 studies. PHCs are the first point of contact of health systems and also the most inclusive. They support health from grassroots level through prevention, education, awareness and treatment initiatives by involving communities and targeting context specific health problems. This setting also interacts with other formal and informal health care settings such as community health centres, households and meeting places to support disease prevention and health promotion efforts in communities. They consist of screening facilities and healthcare resources that are part of the formal health system, thereby serving as an important place for maintaining health of families through equitable and cost effective preventive strategies rather than cost intensive curative strategies [41, 42]. PHC based approaches are also used in providing context specific care to refugees requiring NCD care. Humanitarian shelters face substantial resource constraints and require not only clinical but psychosocial approaches to care as refugees may have experienced volatile conditions for years in their home country and difficult living conditions in the camp. One study mentioned the setting of the NCD intervention in a humanitarian shelter or refugee camp in Jordan housing Syrian refugee families [15]. The multi team based community intervention was specifically conducted in a primary healthcare facility for diabetes and blood pressure control for adult refugees living in the camp or among the general population.

#### People as resources involved

The community resources (people) observed in this setting were primarily nurses and midwives along with community health workers. This however is an observation based on the limited number of studies of the literature review and PHC resources extend beyond nurses and midwives to general practitioners, laboratory technicians and health officers too. Community resources (people) were included to work in coordination for implementation of the programmes and interventions between PHCs and other settings especially community healthcare. They fill important health workforce gaps and understand the needs of the local community facilitating better health outcomes in their area [43]

#### Enabling mechanisms

Task shifting was an important mechanism in this setting. Expanding the practice authority of nurses in PHCs could enable better reach of care and treatment to areas with healthcare accessibility issues. Coordinated working with community health care also enabled increased community support for birth preparedness and enhanced knowledge on pre-eclampsia and other pregnancy related conditions [17]. Coordinated care between PHC and other settings also encourages patients to visit their care provider more regularly and enhanced compliance with medication.

#### Constraining barriers

**Fig 10:**
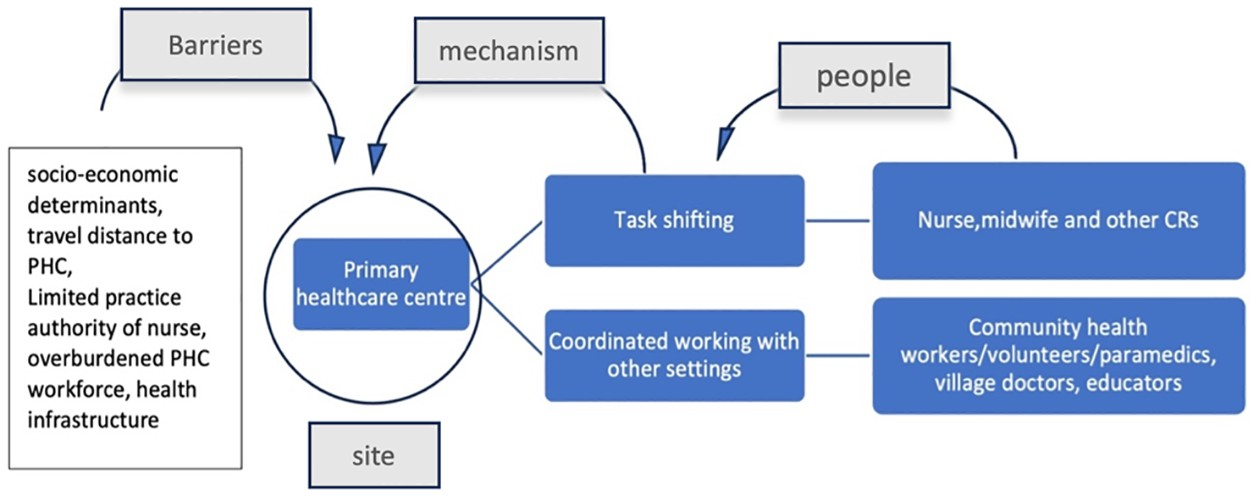
Community resources, enabling mechanisms and barriers associated with the ‘primary healthcare centre’.

Location of PHC, poverty, overburdened health system workforce challenges, health infrastructure, poor quality of PHC facilities and costs of treatment were key barriers observed in this setting. One study discussed practice restrictions on nurses in PHCs as an impediment to diabetes screening for foot ulcers in diabetes management in rural areas of USA [26]. While this study discussed the impact of timely foot ulcer screening in diabetes, NCD management screening for all NCD conditions is a challenge in rural areas and practice limitations on community resources such as nurses, further leads to lack of adequate NCD screening and care in non- urban areas [44] (Fig 10).

### Hospital

One study was conducted in a hospital [33]. Hospital as a setting for NCD prevention and management is useful given its resourcefulness. While hospitals are a part of the secondary and tertiary healthcare system, they do act as settings for integrated care programmes and interventions on diabetes and cardiovascular treatment and management. Hospital resources and networks allow for multifaceted interventions with adequate health infrastructure and monitoring as compared to other settings.

#### People as resources involved

Physicians, patient navigators, health administrators, care coordinators and nurses are resources active in this setting. A USA study conducted in the hospital also included other community based organisations to work in coordination with the hospital to implement the integrated care model for participants enrolled in the study. Patient navigators help facilitate ‘clinical-community linkages’ for patients, thereby making their NCD management journey easier and supported.

#### Enabling mechanism

Key mechanisms enabling successful NCD management programmes in this setting were availability of integrated and personalised care, collaborative environment, equipped care teams, alignment and coordinated health services. Considering the barriers of cost, a value based payment model (where patients pay based on health outcomes rather than number of services or visits) would be more enabling for NCD management interventions in hospitals and clinics [45].

#### Constraining barriers

**Fig 11:**
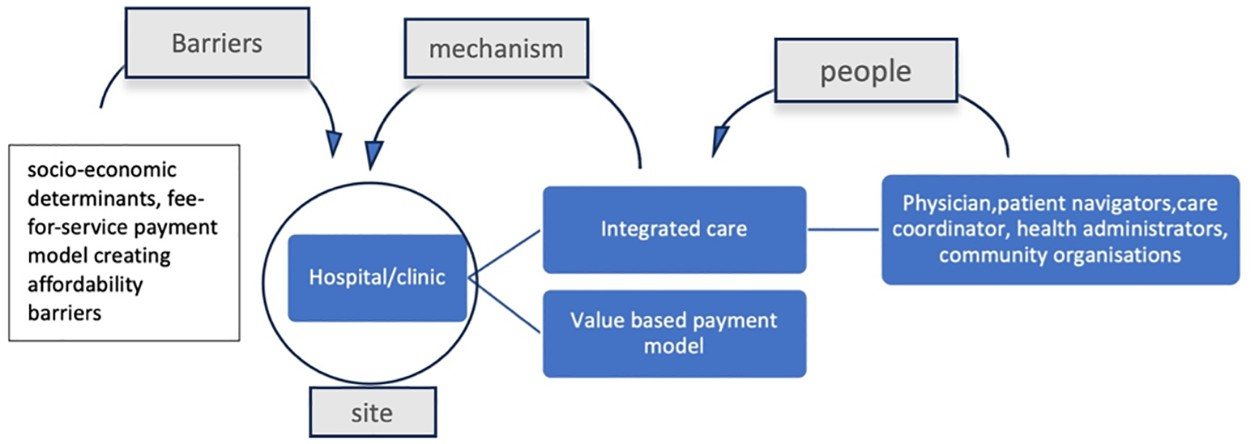
Community resources, enabling mechanisms and barriers associated with the ‘hospital’.

Key barriers in this setting were related to the health system and ‘traditional fee-for-service’ payment model. Fee for service promotes expensive care and unnecessary services which the patients may not require [46], thereby creating affordability barriers to accessing integrated programmes running in hospitals for NCD treatment and management [33] (Fig 11)

### Mobile clinics

One study conducted in South Africa [22], used mobile clinics as a resource for NCD prevention and management in underserved populations who might also need HIV testing. Mobile clinics help reach community resources (people) for NCD prevention and screening services along with other health problems faced by communities and facilitate early diagnosis. Mobile clinics are used in many countries to provide last mile healthcare delivery in hard to reach populations in many countries. They are useful in delivering healthcare where communities may otherwise not travel to health system facilities due to distance, difficult terrains, lack of transportation and affordability issues.

#### People as resources involved

The resources involved in these settings are physicians, nurses, counsellors, health educators, community health workers and the driver. In some cases, the mobile clinics are also equipped with basic laboratory testing and immunisation facilities [47]. Integrated health services through resources in mobile clinics provide integrated and personalised counselling services which maybe more attractive to groups who have privacy concerns or fear of stigma.

#### Enabling mechanism

Mobile clinics are one of the few healthcare community resources that increases the reach of the health system by taking the health system to the community. They offer an entry point for people living in remote areas and unable to access basic health services easily. The mobile clinic acts as a mini clinic and provides integrated care based on community needs. In settings with HIV prevalence where HIV detection is low, mobile clinics provide the benefit of personalised counselling and HIV testing eliminating the barrier of stigma.

#### Constraining barriers

**Fig 12:**
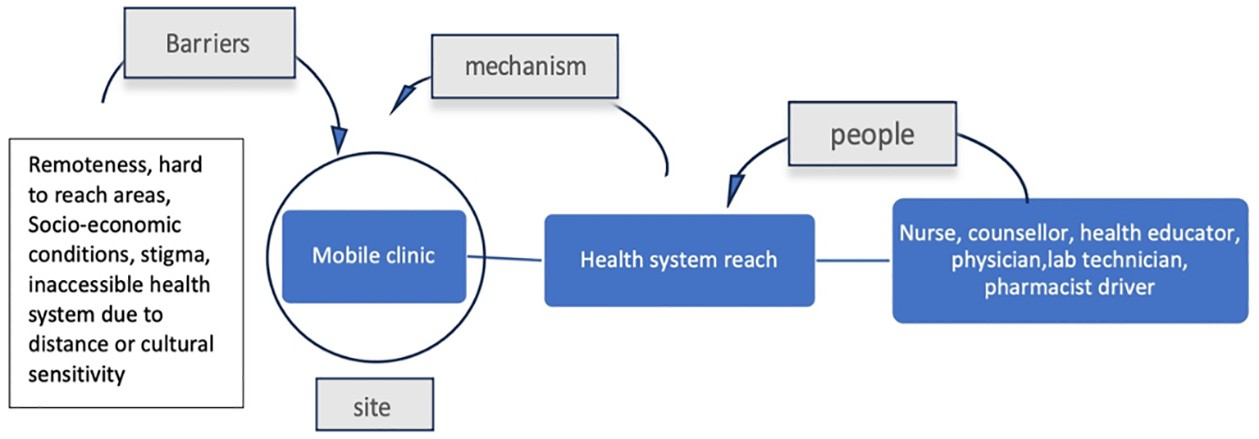
Community resources, enabling mechanisms and barriers associated with the ‘mobile clinic’.

Fragmented healthcare system, economic factors, shame, congested clinics, unfriendly staff, restricted clinic hours and lack of privacy or fear of being seen visiting a physician by the community in areas with high HIV are key barriers to access healthcare. In mobile clinics, retention and scope of long term treatment facilities is an ongoing challenge (Fig 12).

### Pharmacy

One study conducted in Australia used the pharmacy as a community resource [23]. Pharmacies play a crucial role by expanding their role in remote rural areas and conduct screening for NCDs where access to health professionals is not adequate as compared to urban areas. Expanding the role of pharmacies in these areas provide basic screening services to communities through local and collaborative models.

#### People as resources involved

The pharmacist is most important resource in this setting. While there are reservations on expanded role of pharmacists in suggesting treatments, they are capable of provided basic disease testing, vaccinations, spirometry functions, blood glucose and blood pressure measurement. Pharmacists are part of the community they function in and are therefore easily approachable by nearby families for testing and basic health services. Exploring the role of the pharmacist further can also help in creating NCD health awareness initiatives for better information and awareness on health issues.

#### Enabling mechanism

Task shifting is a crucial enabling mechanism for a pharmacy allowing timely detection and basic healthcare access to rural and remote populations. It also has the added benefit of obtaining medication and testing/screening in one place which is known and approachable by community members.

#### Constraining barriers

**Fig 13:**
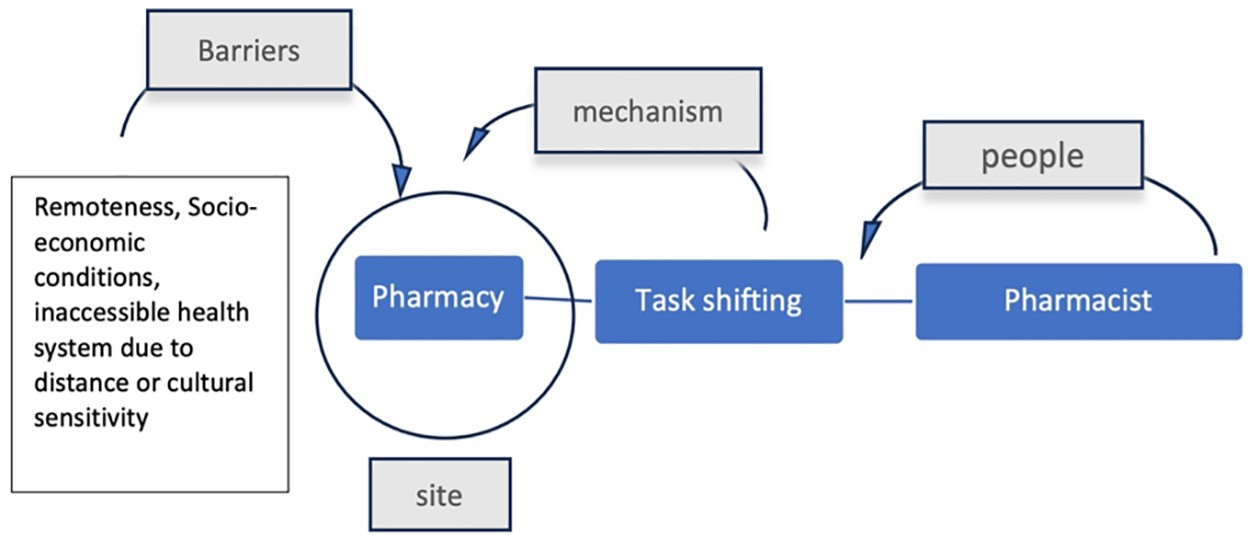
Community resources, enabling mechanisms and barriers associated with the ‘pharmacy’.

This setting can be useful for screening services in remote places where healthcare accessibility is poor due to distances, socio-economic determinants and lack of adequate health workforce. There is a limitation to using the pharmacy as a setting for NCD management. However, prevention activities and screening services are possible under the expanded role of a pharmacist in the local area (Fig 13).

## Discussion

From the findings of the literature sample, seven different sites (’where’) and ten categories of people (’who’) were identified as community resources involved in community based NCD prevention and management interventions. The different types of interventions/actions/descriptions were grouped into eight categories. While these interventions and actions act as enabling mechanisms for creating positive NCD related health outcomes, community resources serve as crucial drivers of the interventions against accessibility barriers posed by health systems and socio-economic conditions.

The ecological model on health promotion [12] touches on two important aspects–(i) the significance of social environmental factors (context) on healthy and unhealthy choices and (ii) the importance of considering context while designing interventions. It suggests that chronic disease prevention and management interventions require a comprehensive approach targeting social environmental factors and not the individual through behaviour change strategies, alone. Since ecological factors or the context, influences health related choices made by individuals in a community, the available community resources (sites and people) and barriers in this conceptual analysis can be considered as important ecological factors influencing NCD related health outcomes alongside the eight categories of mechanisms as community interventions.

The significance of the context as theorised in the ecological model or in this case–site, people and barriers impacting the intervention can be understood by the analysis of simple examples from the sampled literature. Education of family care givers is crucial for diabetes and cardiovascular disease self-management, however, visiting the household [16, 31] to conduct knowledge based programmes through CHWs instead of calling the caregivers to a location for attending a programme- ensured that individuals/caregivers who would otherwise not have attended the programme at a specified location due to time, travel or cost constraints–still obtained important disease management knowledge because of a home based education intervention involving community health workers. Similarly, conducting an integrated programme [33, 35], targeting NCD treatment, management and monitoring along with community-clinical linkages is best facilitated in a hospital setting rather than community centre or primary healthcare centre (or any other setting) because the hospital is better resourced and networked in terms of data monitoring and community organisations. Here, the hospital is helping overcome the barrier of service accessibility by providing NCD treatment, monitoring and community links for support in one place, thereby reducing barriers of complexity of health system navigation by families.

Applying the ecological model to our conceptual analysis, each sphere of influence displays a distinct combination of sites and people based on its context thereby requiring different strategies and combinations of community interventions. The most diverse community resources were being used at interpersonal and community levels, where the interventions were driven by ‘integrated care’ approaches and ‘social support’ by linking patients, community organisations and the health system towards a common goal. The interpersonal level of the ecological model constitutes the formal and informal networks of individuals at home, workplace and their social networks, therefore, interventions based on peer group support and context specific engagement were observed at the interpersonal level involving friends, family caregivers, CHWs, CHVs, peer groups, nurse, midwife, lifecoach, educator, community activator and pharmacist.

At the community level (where interactions between institutions take place), navigators and care coordinators were important community resources in facilitating integrated care interventions. Interactions taking place in ‘integrated care’ interventions involved a more complex network of actors in delivering healthcare e.g. nurses, doctors, health navigators, community organisations, university departments, community paramedics etc., as compared to other mechanisms which involved the informal healthcare work force. The mechanisms involving the formal health system resources were better organised and well resourced. For example, integrated care initiatives linked to the hospital had manpower and capacity to deliver health services in the intervention along with adequately trained workers, while door to door community health initiatives run by CHWs faced challenges in terms of training and financial incentives to continue their programme.

The institutional sites or settings had the most diverse intervention categories as it included important settings such as primary healthcare facilities, mobile clinics and hospitals. It included diverse people as resources like nurse, midwife, physician, CHW, counsellors and health educators. The interventions that worked at this level were community health worker led knowledge dissemination, context specific engagement, personalised and integrated care, reach of health system and task shifting. Even though the institutional sites are formal institutions of the health system, in every study, people as community resources straddled formal institutional settings and informal community settings such as meeting places, community centres. The observation of diverse interventions in the institutional sites may also reflect the capacity of formal health system to mobilise resources from different sectors. Among the most common interventions, community health worker led knowledge dissemination was the only intervention to influence the individuals’ own attitude towards health. Policy level influence was not identified in the sampled literature. None of the community resources (as people) and interventions sought policy change in NCD prevention and management.

Policy was the only sphere of influence in our conceptual analysis where we could not find community interventions and resources. This may be a potential limitation of the analysis as the literature search was derived from a single yet rich database. While we captured a comprehensive set of intervention categories and resources for the prevention and management of chronic diseases, there may be innovative interventions and unidentified resources and barriers especially related to infectious disease and nutritional deficiency, which were not included in the analysis. Alternatively, the identification and categorisation of community resources in this analysis for chronic diseases serves as a starting point to inform the analysis of such resources for other interventions and health conditions. The evolution of the conceptual analysis into a unique community resource framework, is rooted in its authors’ professional and lived experience of chronic disease prevention and management–a strength of the framework to be open to draw on and be developed by future research and studies.

Notably, this conceptual analysis is about the prevention and management of NCDs within the community, and so is inevitably focussed on the primary healthcare level as the first and most inclusive point of contact in the health system. We are therefore unable to provide a comprehensive categorisation across levels of care. However, the conceptual framework we have developed provides a structured template for future research to draw on and develop. Categorisation at the secondary and tertiary systems would definitely lead to important insights. But we recognise that such categorisation may be limited to ‘treatment’ of NCDs and integrated health delivery services. In addition, we note the lack of community resources and health interventions invested in directly influencing health policy through advocacy, this may be considered a gap between perceived needs (in the community) and responsibility for addressing them (by governments). This observation can be useful in beginning to think about how the role of health resources closest to ground can be leveraged to inform policies of health systems.

## Conclusion

This conceptual analysis focussed on identifying and classifying different community resources and community interventions/programmes/descriptions/actions (enabling mechanisms) for prevention and management of type two diabetes and cardiovascular disease. In the process, we also recognised the importance of context and barriers that influence the setting in which these interventions take place thereby allowing us to map the intervention categories to relevant levels of the social-ecological model [12] and understand better, regarding which interventions and resources work in which sphere of influence. While this analysis is limited in its scope, the interpretations and inter-relationships identified can be useful in (i) determining existing approaches in NCD interventions using community resources (ii) helping explore and understand commonalities between different community resources and interventions to innovate and replicate good practices (iii) understanding inter-relationships between various community resources, mechanisms, barriers and settings against their context and inform future interventions in NCD prevention and management.

## Supporting information

S1 Text List of studies included the literature review. (PDF)

S1 Checklist PRISMA checklist. (DOCX)
