# Supplementary material for: People and sites as community resources for preventing and managing chronic health conditions: A conceptual analysis
Source: PLOS Glob Public Health. 2024 Jul 26;4(7):e0003415. doi: 10.1371/journal.pgph.0003415 (PMC11280530; doi:10.1371/journal.pgph.0003415)
Supplement: S1 Text — (PDF) [file pgph.0003415.s001.pdf]

Article	Reference
"Everything in One Place": Stakeholder Perceptions of Integrated Medical and Social Care for Diabetes Patients in Western Maryland.	Saulsberry, L., Gunter, K. E., O'Neal, Y., Tanumihardjo, J., Gauthier, R., Chin, M. H., & Peek, M. E. (2023). "Everything in One Place": Stakeholder Perceptions of Integrated Medical and Social Care for Diabetes Patients in Western Maryland. Journal of General Internal Medicine, 38(1), 25-32. https://doi.org/10.1007/s11606-022-07919-1
Assessing the impact of community-based interventions on hypertension and diabetes management in three Minnesota communities: Findings from the prospective evaluation of US HealthRise programs.	Fullman, N., Cowling, K., Flor, L. S., Wilson, S., Bhatt, P., Bryant, M. F., Camarda, J. N., Colombara, D. V., Daly, J., Gabert, R. K., Harris, K. P., Johanns, C. K., Mandile, C., Marshall, S., McNellan, C. R., Mulakaluri, V., Phillips, B. K., Reitsma, M. B., Sadighi, N., . . . Gakidou, E. (2023). Assessing the impact of community-based interventions on hypertension and diabetes management in three Minnesota communities: Findings from the prospective evaluation of US HealthRise programs. PLOS ONE, 18(2), e0279230. https://doi.org/10.1371/journal.pone.0279230
Peer support facilitator and peer perspectives of an inner-regional Australian diabetes prevention and self-management peer support program.	Kim, C., MacMillan, F., Osuagwu, U. L., & Simmons, D. (2022). Peer support facilitator and peer perspectives of an inner-regional Australian diabetes prevention and self-management peer support program. Health & Social Care in the Community, 30(6), e4051-e4064. https://doi.org/https://doi.org/10.1111/hsc.13798

Community perspectives on cardiovascular disease control in rural Ghana: A qualitative study.	Patil, B., Hutchinson Maddox, I., Aborigo, R., Squires, A. P., Awuni, D., Horowitz, C. R., Oduro, A. R., Phillips, J. F., Jones, K. R., & Heller, D. J. (2023). Community perspectives on cardiovascular disease control in rural Ghana: A qualitative study. PLOS ONE, 18(1), e0280358. https://doi.org/10.1371/journal.pone.0280358
Community engagement for birth preparedness and complication readiness in the Community Level Interventions for Pre-eclampsia (CLIP) Trial in India: a mixed-method evaluation.	Kavi, A., Kinshella, M.-L. W., Ramadurg, U. Y., Charantimath, U., Katageri, G. M., Karadiguddi, C. C., Honnungar, N. V., Bannale, S. G., Mungarwadi, G. I., Bone, J. N., Vidler, M., Magee, L., Mallapur, A., Goudar, S. S., Bellad, M., Derman, R., Dadelszen, P. v., & Group, T. C. I. W. (2022). Community engagement for birth preparedness and complication readiness in the Community Level Interventions for Pre-eclampsia (CLIP) Trial in India: a mixed-method evaluation. BMJ Open, 12(12), e060593. https://doi.org/10.1136/bmjopen-2021-060593
Bridge to Health/ Puente a la Salud: Rationale and design of a pilot feasibility randomized trial to address diabetes self-management and unmet basic needs among racial/ethnic minority and low-income patients.	Bridge to Health/ Puente a la Salud: Rationale and design of a pilot feasibility randomized trial to address diabetes self-management and unmet basic needs among racial/ethnic minority and low-income patients.

Care Partner Support Following a Diabetes Self-Management Education and Support Intervention.	Papajorgji-Taylor, D., Francisco, M., Schneider, J. L., Vaughn, K., Lindberg, N., Smith, N., & Fitzpatrick, S. L. (2021). Bridge to Health/ Puente a la Salud: Rationale and design of a pilot feasibility randomized trial to address diabetes self-management and unmet basic needs among racial/ethnic minority and low-income patients. Contemporary Clinical Trials Communications, 22, 100779. https://doi.org/https://doi.org/10.1016/j.conctc.2021.100779
Group Social Support Facilitates Adoption of Healthier Behaviors Among Black Women in a Community-Initiated National Diabetes Prevention Program.	O'Neal, L. J., Scarton, L., & Dhar, B. (2022). Group Social Support Facilitates Adoption of Healthier Behaviors Among Black Women in a Community-Initiated National Diabetes Prevention Program. Health Promot Pract, 23(6), 916-919. https://doi.org/10.1177/15248399211045989
Nurse practitioner scope of practice and the prevention of foot complications in rural diabetes patients.	Hughes, D. R., Filar, C., & Mitchell, D. T. (2022). Nurse practitioner scope of practice and the prevention of foot complications in rural diabetes patients. The Journal of Rural Health, 38(4), 994-998. https://doi.org/https://doi.org/10.1111/jrh.12599
The Adaptation of a Youth Diabetes Prevention Program for Aboriginal Children in Central Australia: Community Perspectives.	Rohit, A., McCarthy, L., Mack, S., Silver, B., Turner, S., Baur, L. A., Canuto, K., Boffa, J., Dabelea, D., Sauder, K. A., Maple-Brown, L., & Kirkham, R. (2021). The Adaptation of a Youth Diabetes Prevention Program for

	Aboriginal Children in Central Australia: Community Perspectives. Int J Environ Res Public Health, 18(17). https://doi.org/10.3390/ijerph18179173
Management of diabetes mellitus at the household level using community health strategy in Embu County, Kenya.	Hussein, S. A., Kithuka, P., Otieno, G., Yoos, A., Kaugi, R., & Njeru, C. (2021). Management of diabetes mellitus at the household level using community health strategy in Embu County, Kenya. Pan Afr Med J, 39, 35. https://doi.org/10.11604/pamj.2021.39.35.27753
The role of the Basic Public Health Service program in the control of hypertension in China: Results from a cross-sectional health service interview survey.	Qin, J., Zhang, Y., Fridman, M., Sweeny, K., Zhang, L., Lin, C., & Mao, L. (2021). The role of the Basic Public Health Service program in the control of hypertension in China: Results from a cross-sectional health service interview survey. PLOS ONE, 16(6), e0217185. https://doi.org/10.1371/journal.pone.0217185
Reaching underserved South Africans with integrated chronic disease screening and mobile HIV counselling and testing: A retrospective, longitudinal study conducted in Cape Town.	Smith, P. J., Davey, D. J., Green, H., Cornell, M., & Bekker, L.-G. (2021). Reaching underserved South Africans with integrated chronic disease screening and mobile HIV counselling and testing: A retrospective, longitudinal study conducted in Cape Town. PLOS ONE, 16(5), e0249600. https://doi.org/10.1371/journal.pone.0249600
A funfair without the candy floss: engaging communities to prevent diabetes in Nepal.	Morrison, J., & Arjyal, A. (2021). A funfair without the candy floss: engaging communities to prevent diabetes in Nepal. Public Health, 193, 23-25.

	https://doi.org/https://doi.org/10.1016/j.puhe.2021.01.012
Rationale and Design of a Cluster Randomized Trial of a Village Doctor-Led Intervention on Hypertension Control in China.	Sun, Y., Li, Z., Guo, X., Zhou, Y., Ouyang, N., Xing, L., Sun, G., Mu, J., Wang, D., Zhao, C., Wang, J., Ye, N., Zheng, L., Chen, S., Chang, Y., Yang, R., He, J., & Group, C. S. (2021). Rationale and Design of a Cluster Randomized Trial of a Village Doctor-Led Intervention on Hypertension Control in China. American Journal of Hypertension, 34(8), 831-839. https://doi.org/10.1093/ajh/hpab038
Clinical outcomes in a primary-level non-communicable disease programme for Syrian refugees and the host population in Jordan: A cohort analysis using routine data.	Ansbro, É., Homan, T., Prieto Merino, D., Jobanputra, K., Qasem, J., Muhammad, S., Fardous, T., & Perel, P. (2021). Clinical outcomes in a primary-level non-communicable disease programme for Syrian refugees and the host population in Jordan: A cohort analysis using routine data. PLOS Medicine, 18(1), e1003279. https://doi.org/10.1371/journal.pmed.1003279
Shared medical appointments for Innu patients with well-controlled diabetes in a Northern First Nation Community.	Karaivanov, Y., Philpott, E. E., Asghari, S., Graham, J., & Lane, D. M. (2021). Shared medical appointments for Innu patients with well-controlled diabetes in a Northern First Nation Community. Can J Rural Med, 26(1), 19-27. https://doi.org/10.4103/cjrm.Cjrm_45_20
Community Medication Education, Data, & Safety (C-MEDS): Findings from a Pilot Project.	Meyer, M., Enguidanos, S., Zhu, Y., Likar, D., & Batra, R. (2021). Community Medication Education, Data, & Safety (C-MEDS): Findings from a Pilot Project. Journal

	of the American Geriatrics Society, 69(3), 813-821. https://doi.org/https://doi.org/10.1111/jgs.16981
Early detection of type 2 diabetes in socioeconomically disadvantaged areas in Stockholm - comparing reach of community and facility-based screening.	Timm, L., Harcke, K., Karlsson, I., Sidney Annerstedt, K., Alvesson, H. M., Stattin, N. S., Forsberg, B. C., Östenson, C.-G., & Daivadanam, M. (2020). Early detection of type 2 diabetes in socioeconomically disadvantaged areas in Stockholm – comparing reach of community and facility-based screening. Global Health Action, 13(1), 1795439. https://doi.org/10.1080/16549716.2020.1795439
Health professional perspectives of expanded practice in rural community pharmacy in Australia.	Taylor, S., Cairns, A., & Glass, B. (2020). Health professional perspectives of expanded practice in rural community pharmacy in Australia. International Journal of Pharmacy Practice, 28(5), 458-465. https://doi.org/10.1111/ijpp.12648
Community intervention for cardiovascular disease risk factors in Kalutara, Sri Lanka.	Gamlath, L., Nandasena, S., Silva, P., Morrell, S., Linhart, C., Lin, S., Sharpe, A., Nathan, S., & Taylor, R. (2020). Community intervention for cardiovascular disease risk factors in Kalutara, Sri Lanka. BMC Cardiovasc Disord, 20(1), 203. https://doi.org/10.1186/s12872-020-01427-y
Strengthening community-clinical linkages to reduce cardiovascular disease risk in rural NC: feasibility phase of the CHANGE study.	Samuel-Hodge, C. D., Gizlice, Z., Allgood, S. D., Bunton, A. J., Erskine, A., Leeman, J., & Cykert, S. (2020). Strengthening community-clinical linkages to reduce cardiovascular disease risk in rural NC: feasibility phase

	of the CHANGE study. BMC Public Health, 20(1), 264. https://doi.org/10.1186/s12889-020-8223-x
An Emerging Model for Community Health Worker-Based Chronic Care Management for Patients With High Health Care Costs in Rural Appalachia.	Crespo, R., Christiansen, M., Tieman, K., & Wittberg, R. (2020). An Emerging Model for Community Health Worker-Based Chronic Care Management for Patients With High Health Care Costs in Rural Appalachia. Prev Chronic Dis, 17, E13. https://doi.org/10.5888/pcd17.190316
Social networks, perceived social support, and HbA1c in individuals with type 2 diabetes mellitus in urban Ghana.	Botchway, M., Davis, R. E., Merchant, A. T., Appiah, L. T., Sarfo-Kantanka, O., & Moore, S. (2023). Social networks, perceived social support, and HbA1c in individuals with type 2 diabetes mellitus in urban Ghana. Ethnicity & Health, 28(2), 281-298. https://doi.org/10.1080/13557858.2022.2033172
